# Plant-Derived Bioactives in Tendon and Enthesis Biology: An Evidence-Tiered Narrative Review

**DOI:** 10.3390/nu18132120

**Published:** 2026-06-30

**Authors:** Dojoon Park, Hae-Seok Koh, Youn-Ho Choi, Keun-Kyoung Kim, Ilkyu Park

**Affiliations:** 1 Department of Orthopaedic Surgery, St. Vincent’s Hospital, College of Medicine, The Catholic University of Korea, Suwon 16247, Republic of Korea; onedream1106@naver.com (D.P.); vincentos@naver.com (H.-S.K.); kkieek@hanmail.net (Y.-H.C.); inniken285@gmail.com (K.-K.K.); 2 Department of Orthopaedic Surgery, Bucheon St. Mary’s Hospital, College of Medicine, The Catholic University of Korea, Bucheon 14647, Republic of Korea

**Keywords:** nutraceuticals, rotator cuff, enthesis, senescence, bioavailability, formulation-specific evidence, tendon–bone healing, dietary bioactives

## Abstract

Tendon injury, tendon–bone interface disruption, and rotator cuff pathology involve inflammatory signaling, oxidative stress, extracellular matrix turnover, cellular senescence, and impaired differentiation. Plant-derived bioactive compounds, including curcumin, quercetin, and other flavonoids and botanical formulations, have been investigated across these pathways in preclinical tendon and enthesis models, but interpretation is complicated by heterogeneous models, formulations, delivery platforms, and endpoints. This review distinguishes preclinical and formulation-specific tissue-response signals from evidence sufficient to support patient-facing clinical or nutraceutical claims. It synthesizes 31 articles using an evidence-tiering framework separating preclinical plausibility (Tier 3), limited human-facing translational evidence (Tier 2), and robust clinical efficacy (Tier 1). The corpus was predominantly Tier 3, with two Tier 2 articles providing human tissue or clinically oriented evidence and no Tier 1 evidence identified. An evidence-calibrated translational proximity map and an overclaim prevention checklist are provided to guide interpretation. The proposed proximity map reflects relative translational position within a focused narrative corpus and was not based on systematic review methods, formal risk-of-bias assessment, or clinical intervention evidence. The limited human-facing evidence does not represent patient-intervention evidence and does not support clinical, supplementation, or treatment recommendations. These compounds remain candidates for staged translational investigation rather than established interventions.

## 1. Introduction

Tendon injuries, rotator cuff tears, and tendon–bone interface disruptions are common clinical problems characterized by limited intrinsic healing capacity, high recurrence rates, and prolonged functional recovery [1]. These tissues—tendon, enthesis, and the peritendinous environment—differ in cellularity, vascularity, and mechanical demands yet share overlapping biological stress responses: chronic inflammation, oxidative injury, extracellular matrix dysregulation, cellular senescence, aberrant differentiation, and fatty infiltration [2,3,4]. Understanding these shared pathways at the tissue level is a prerequisite for evaluating any candidate intervention, whether pharmacologic, biologic, or biomaterial based.

Plant-derived bioactive compounds have attracted interest in this context. Chemically diverse molecules—polyphenols such as curcumin and quercetin flavonoids such as baicalein and icariin, terpenoids such as tanshinone IIA, ginsenosides, and various botanical extracts—have been investigated for activities intersecting with inflammatory, redox, senescence-associated, and matrix-regulatory pathways in tendon and tendon–bone interface models [5,6,7,8,9,10,11]. However, these compounds differ substantially in source, structure, bioavailability, and delivery context. A curcumin-loaded hydrogel, a quercetin-containing aligned nanowire scaffold, and oral ginsenoside administration represent fundamentally different interventions despite sharing the broad label of “plant-derived bioactives.” Interpreting such evidence requires compound- and formulation-specific assessment rather than class-wide generalization.

Despite growing interest in plant-derived bioactive compounds for tendon, rotator cuff, and tendon–bone interface biology, the current literature remains fragmented across compound classes, delivery platforms, tissue models, and mechanistic endpoints. More importantly, the boundary between preclinical plausibility and clinical relevance is often insufficiently defined. Findings derived from animal models, in vitro systems, ex vivo tissue exposure, hydrogels, scaffolds, nanomicelles, or combination interventions may be difficult to interpret when they are generalized to compound-level, oral nutraceutical, or patient-facing claims. Thus, the critical gap is not simply the absence of additional preclinical studies but the lack of an evidence-tiered framework that defines what the current evidence can reasonably support.

**Figure 1:**
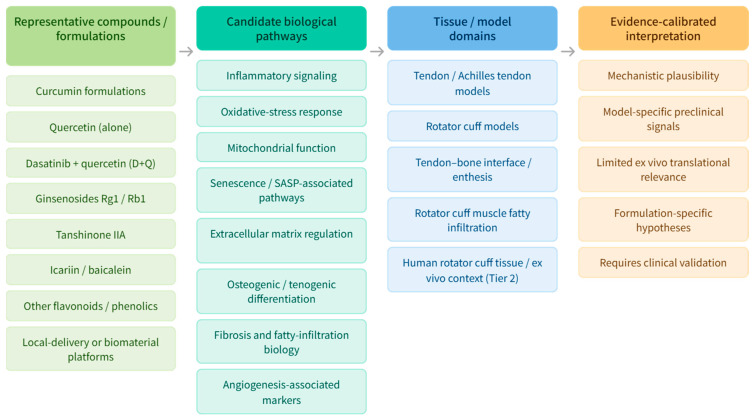
Conceptual framework linking representative plant-derived bioactive compounds to candidate biological pathways in tendon and tendon–bone interface biology. Compounds and formulations are mapped to inflammatory signaling, oxidative-stress response, cellular senescence, extracellular matrix regulation, lineage differentiation, and fatty-infiltration/fibrosis pathways. Arrows indicate preclinical associations and model-specific observations, not clinical efficacy. D + Q is shown separately from quercetin-only evidence. Formulation-specific findings are labeled as platform-dependent.

To address this gap, the present review applies an evidence-tiering framework to a focused 31-article corpus on plant-derived bioactive compounds in tendon and tendon–bone interface biology. Beyond cataloguing candidate mechanisms (Figure 1), this review provides two practical outputs: an evidence-calibrated translational proximity map of compound–pathway–tissue combinations and an overclaim prevention checklist that maps common interpretive scenarios to appropriate claim boundaries. The aim is not to establish clinical efficacy, but to clarify the current translational frontier, distinguish formulation-specific and combination-intervention findings from compound-level claims, and identify the validation steps required before patient-facing conclusions can be justified. This focus is directly relevant to the nutrition and nutraceutical community, where preclinical findings involving plant-derived compounds are sometimes extrapolated to dietary supplementation or functional-food claims without sufficient clinical validation. By defining claim boundaries at the formulation, model, and evidence-tier levels, this review provides a translational filter against premature nutraceutical positioning of compounds whose evidence remains preclinical and delivery-specific.

## 2. Methods: Focused Narrative Synthesis and Evidence-Boundary Framework

### 2.1. Review Design

This manuscript is a structured narrative review of plant-derived bioactive compounds in tendon and tendon–bone interface biology, with emphasis on mechanistic and translational synthesis. It was not designed as a systematic review or meta-analysis; accordingly, it does not claim PRISMA compliance, does not pool effect sizes, and does not attempt to determine clinical treatment efficacy. This review was designed to map mechanistic signals, translational proximity, and claim boundaries rather than to estimate clinical efficacy.

### 2.2. Evidence Source and Corpus Construction

The working evidence base comprised 31 articles relevant to plant-derived bioactive compounds in tendon injury, rotator cuff pathology, tendon–bone interface biology, and related translational mechanisms. To construct a focused evidence corpus rather than an exhaustive systematic dataset, targeted searches were conducted in PubMed/MEDLINE, Scopus, and Google Scholar for English-language, peer-reviewed articles published between January 2016 and May 2026; the last search was performed in May 2026.

Searches combined compound-related terms with tissue- and mechanism-related terms. Compound-related terms included curcumin, quercetin, baicalein, icariin, tanshinone IIA, ginsenosides, Rhizoma drynariae, farnesol, procyanidins, caffeic acid, protocatechuic acid, and botanical extract. Tissue- and mechanism-related terms included tendon, Achilles tendon, rotator cuff, tendon–bone healing, enthesis, tendon adhesion, senescence, oxidative stress, inflammation, extracellular matrix, fatty infiltration, hydrogel, scaffold, and biomaterial delivery. A representative PubMed/MEDLINE search string was: (“curcumin” OR “quercetin” OR “baicalein” OR “icariin” OR “tanshinone IIA” OR “ginsenoside” OR “Rhizoma drynariae” OR “farnesol” OR “procyanidin” OR “caffeic acid” OR “protocatechuic acid” OR “botanical extract”) AND (“tendon” OR “Achilles tendon” OR “rotator cuff” OR “tendon–bone” OR “enthesis” OR “tendinopathy”). Analogous TITLE-ABS-KEY queries were used in Scopus. In Google Scholar, the same compound-tissue term combinations were entered, results were sorted by relevance, and the titles and abstracts of the most relevant records were screened on a per-query basis until further records no longer met the eligibility criteria. Because Google Scholar results are dynamically ranked and not fully reproducible, this source was used as a supplementary sensitivity search rather than as a source for quantitative record counting. Reference lists of relevant reviews and key primary studies were additionally screened to identify further eligible sources.

Sources were eligible for core synthesis when they (1) evaluated a plant-derived compound, botanical extract, or plant-associated formulation; (2) used a tendon, rotator cuff, tendon–bone interface, or closely related tissue model; and (3) reported mechanism-linked molecular, histologic, biomechanical, cellular, or tissue-response outcomes. Sources were excluded from core synthesis when they did not involve a plant-derived component, did not use a tendon, rotator cuff, or tendon–bone interface model, did not report mechanism-linked outcomes, or were not available as peer-reviewed English-language full texts. Studies outside the target tissue domain were not used as core evidence and were retained only as Tier 4 background sources when they clarified pathway-level interpretation. Dasatinib plus quercetin (D + Q) was retained because quercetin is the plant-derived component of the combination and because this evidence provides the only human tissue-level translational data currently available for a quercetin-associated intervention in tendon biology within the reviewed corpus; however, D + Q findings were interpreted throughout as combination-specific evidence and were not attributed to quercetin alone.

Searching, initial screening, and data extraction were performed by the lead author (D.P.); the resulting corpus, evidence-tier assignments, and claim-boundary interpretations were independently reviewed and verified by the co-authors responsible for literature curation (Y.-H.C. and K.-K.K.), with disagreements resolved by consensus under the supervision of the senior author (I.P.). The final 31-article corpus was not predetermined; it resulted from applying the focused eligibility criteria above to the targeted search output and reference-list screening. Because this was a focused, non-exhaustive narrative search rather than a PRISMA-based systematic review, complete database-yield counts (records identified and excluded at each stage) were not logged during the original search process. Supplementary Figure S1 therefore depicts the corpus-prioritization logic together with the available numerical information, including the final 31-article corpus and its tier distribution, rather than a PRISMA-style record-flow diagram. This strategy was intended to support transparent evidence mapping, claim calibration, and translational prioritization within a focused corpus, not to claim exhaustive capture of all eligible studies.

### 2.3. Evidence Extraction

Each article was processed using a structured extraction framework. The core tabulated fields, reported for all 31 articles in Supplementary Table S1, were: compound or formulation identity, plant-derived component, non-plant component where applicable, target tissue or model domain, study type, evidence tier, key mechanism or endpoint, and the principal translational limitation. Dosing, exposure duration, and safety observations were recorded narratively when reported by the source article but were not uniformly available across the corpus and were therefore not tabulated as complete fields; this limitation is addressed in Section 6.2. For each source, the framework explicitly separated what the article directly supported from what could not be inferred, with particular attention to overinterpretation risks including unsupported clinical extrapolation, regeneration language, and inappropriate generalization from formulation-specific studies.

### 2.4. Evidence Tiering

Established evidence-grading frameworks, such as GRADE or the Oxford Centre for Evidence-Based Medicine levels of evidence, are primarily designed to evaluate clinical certainty or intervention efficacy in patient-centered research. Because the present corpus is dominated by preclinical, mechanistic, ex vivo, and formulation-specific studies without clinical intervention data, a simplified four-tier framework was adopted to classify evidence by its distance from patient-facing validation rather than by clinical certainty. Each article was assigned to one of four evidence tiers:

Tier 1: Robust clinical evidence, such as high-quality randomized controlled trials or meta-analyses evaluating clinical efficacy.

Tier 2: Moderate clinical or translational human-facing evidence, including human tissue, ex vivo, or clinically oriented review evidence without RCT-level efficacy support.

Tier 3: Preclinical or mechanistic evidence, including animal, in vitro, biomaterial, network pharmacology, and controlled laboratory studies.

Tier 4: Indirect, off-topic, or limited-use evidence not suitable as core support for tendon or tendon–bone efficacy claims.

Among the 31 articles, no Tier 1 evidence was identified. Two articles were classified as Tier 2, 27 as Tier 3, and two as Tier 4. The evidence base is therefore dominated by preclinical and mechanistic studies. Evidence tiers were used not only to summarize study type but also to define the ceiling of permissible claims for each source.

The four-tier framework was defined a priori, before evidence extraction, to provide a consistent basis for classifying each source by its distance from patient-facing validation. Tier assignments were applied by the lead author and independently verified by the curation co-authors as described in Section 2.2, with disagreements resolved by consensus. It should be emphasized that this framework classifies distance from patient-facing validation rather than internal validity, methodological quality, or certainty of treatment effect; a Tier 3 designation therefore reflects preclinical or formulation-specific status, not a judgment of study rigor. No formal risk-of-bias assessment or certainty grading (e.g., GRADE) was performed.

### 2.5. Narrative Synthesis

Evidence was synthesized by biological mechanism, compound class, tissue/model context, and translational applicability. Mechanistic categories included inflammatory signaling, oxidative stress, senescence-associated pathways, extracellular matrix regulation, lineage differentiation, fatty infiltration, and tissue-interface biology. Formulation-specific findings were interpreted according to the tested delivery context and were not generalized across preparations.

### 2.6. Claim-Boundary and Interpretive Conventions

Throughout this review, the following interpretive principles apply to all sections without repeated statements:

Preclinical and mechanistic findings are described using bounded language (e.g., “was associated with,” “may modulate,” “supports mechanistic plausibility”). Formulation-specific findings (hydrogels, scaffolds, nanomicelles, membranes, controlled-release systems) are not generalized to oral supplementation, dietary exposure, or compound-only effects. Combination interventions (e.g., dasatinib plus quercetin) are interpreted as combination-specific evidence and are not attributed to individual components. Structural, histologic, biomechanical, and molecular indices in animal models are not interpreted as patient-level symptomatic benefit, reduced surgical failure, or clinical recovery. Disease-modifying, regenerative, and treatment-recommendation language is avoided unless directly supported by the source evidence.

The full compound-level and tissue-level evidence base is summarized in Table 1 and Table 2, which are discussed in detail in the synthesis that follows. These conventions establish the claim ceiling for the entire manuscript. Table 3 operationalizes these principles as an overclaim prevention checklist that links common interpretive scenarios to appropriate claim boundaries, and it is proposed as a practical interpretive tool for calibrating claim boundaries in preclinical, formulation-specific, and combination-intervention evidence. Readers should assume bounded interpretation throughout, even where cautionary language is not individually restated.

## 3. Evidence Landscape: Compounds, Models, and Tier Distribution

### 3.1. Compound Classes

The corpus includes several broad compound categories, each represented by distinct preparations and model contexts. Polyphenol- and flavonoid-related compounds are the most frequently represented group: curcumin appears across multiple tendon-related animal models and formulation systems [5,8,12,14,15,16,17]; quercetin-only evidence spans aged tendon, tendinopathy, and tendon stem/progenitor cell models [7,18,19,20]; baicalein and icariin have been studied in tendon–bone interface contexts [6,9]; and total flavonoids of Rhizoma drynariae (TFRD) appear in ACL reconstruction models [23,24]. Phenolic acids, including protocatechuic acid and caffeic acid, provide limited single-study evidence [25,26].

Terpenoid-related compounds include tanshinone IIA, studied in rotator cuff fatty-infiltration and Achilles tendon models [2,11,22], and farnesol, investigated as a local hydrogel membrane in a rotator cuff model [28]. Ginsenosides Rg1 and Rb1 have been examined in Achilles tendinitis and rotator cuff fibrosis contexts [3,10].

These categories are organizational, not biologically uniform. Compounds within the same class differ in source, bioavailability, delivery route, and measured outcome. Table 1 summarizes the compound-level evidence characteristics, emphasizing that each compound should be interpreted according to its tested formulation, route, tissue model, and evidence tier.

### 3.2. Model Systems and Tissue Contexts

Evidence is concentrated in three overlapping domains: Achilles tendon injury and remodeling models (the largest subgroup); rotator cuff tear biology, including fatty infiltration and senescence; and tendon–bone interface models spanning rotator cuff reconstruction and ACL-related contexts. Formulation-specific studies—including hydrogels, scaffolds, nanomicelles, membranes, and controlled-release systems—constitute a substantial portion of the corpus and are interpreted as delivery-platform evidence rather than compound-only evidence. Table 2 maps these evidence clusters by tendon, rotator cuff, tendon–bone interface, and related tissue-domain contexts, showing where the current corpus is most concentrated and how each domain should be interpreted translationally.

### 3.3. Tier Distribution and Claim Ceiling

**Figure 2:**
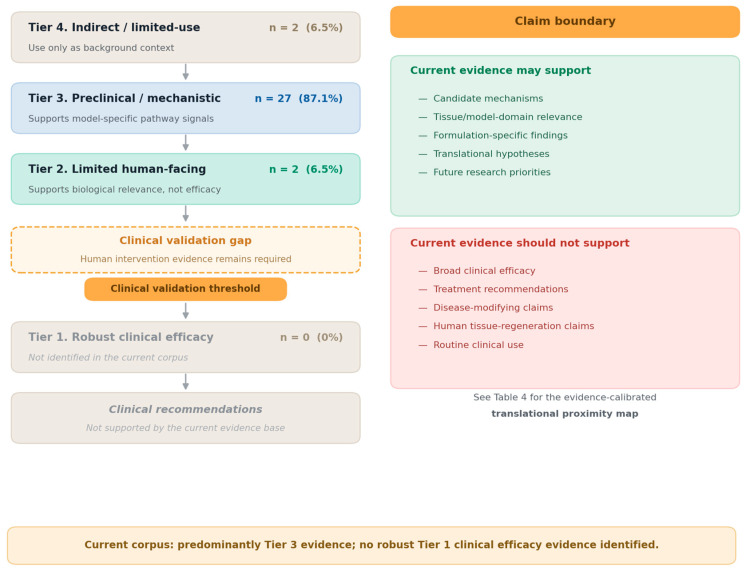
Evidence hierarchy and claim boundaries. Evidence tiers reflect the 31-article corpus. Tier distribution was Tier 1 = 0/31 (0%), Tier 2 = 2/31 (6.5%), Tier 3 = 27/31 (87.1%), and Tier 4 = 2/31 (6.5%). No Tier 1 robust clinical efficacy evidence was identified. Most evidence is Tier 3 preclinical or mechanistic. Tier 2 evidence is limited to one human tissue/ex vivo D + Q study and one curcumin scoping review. The figure defines the claim boundary: preclinical findings support pathway-level hypotheses; human tissue/ex vivo findings support translational relevance; clinical efficacy requires controlled human intervention studies not yet available in this corpus. Arrows indicate progression across evidence tiers and the translational gap toward clinical validation; colors distinguish evidence levels and claim-boundary categories; the dashed box highlights the clinical validation gap between limited human-facing evidence and robust clinical efficacy.

The tier distribution defines the interpretive ceiling for this review. With 27 Tier 3 articles, two Tier 2 sources, and no Tier 1 evidence, the corpus supports discussion of candidate mechanisms, model-specific pathway signals, and translational hypotheses. The two Tier 2 sources—a human rotator cuff tissue/ex vivo study of dasatinib plus quercetin [4] and a curcumin-focused scoping review [13]—provide translational context but not patient-intervention evidence. Figure 2 illustrates this evidence hierarchy.

## 4. Biological Mechanisms in Tendon and Tendon–Bone Interface Models

### 4.1. Inflammatory Signaling and Cytokine-Related Pathways

Curcumin was associated with reduced peritendinous inflammation and adhesion formation in rat tendon models [5,14,17], while a scoping review discussed NF-κB-related signaling in tendinopathy [13]. Farnesol delivered via gellan gum/hyaluronic acid hydrogel membrane attenuated IL-6 and TNF-α expression in a rabbit rotator cuff model [28]. Quercetin reduced inflammatory, apoptotic, and MMP expression markers in a collagenase-induced rat Achilles tendinopathy model [7]. Ginsenoside Rg1 modulated inflammatory signaling through IGF1R/estrogen receptor pathways in a rat Achilles tendinitis model [10], and tanshinone IIA showed anti-inflammatory effects in both Achilles tendon [11] and rotator cuff fatty-infiltration contexts [2].

Across these studies, inflammatory pathway modulation is the most recurrent signal. However, measured endpoints vary considerably—ranging from cytokine expression and NF-κB activity to histologic inflammation scoring—which limits direct cross-study comparison. Greater endpoint standardization would help determine whether the convergent signals across compounds reflect shared anti-inflammatory mechanisms or distinct pathway interactions.

### 4.2. Oxidative Stress Response and Mitochondrial Function

Oxidative-stress biology intersects with tendon and rotator cuff pathology through ROS accumulation, antioxidant enzyme activity, and mitochondrial dysfunction. Curcumin was associated with changes in SOD, MDA, and related oxidative-stress markers alongside collagen and biomechanical improvements in rat Achilles tendon models [8,16]. Quercetin modulated oxidative-stress endpoints in both tendinopathy [7] and aged tendon stem/progenitor cell contexts, with the latter involving mitophagy-related mechanisms via AKT/NF-κB/NLRP3 signaling [20]. Protocatechuic acid showed antioxidant activity in a chronic rotator cuff animal model, although several outcomes were nonsignificant [26]. Ginsenoside Rb1 evidence included mitochondrial Ca^2+^-related markers in rotator cuff fibrosis and fatty-infiltration models [3].

Mitochondrial function is less broadly represented than inflammatory or matrix endpoints, but the convergence of quercetin-mitophagy and Rb1-mitochondrial signals suggests organelle-level stress as a candidate pathway worth further investigation, particularly in age-related tendon biology.

### 4.3. Extracellular Matrix Regulation and Collagen Organization

Matrix-related findings derive primarily from tendon and tendon–bone interface models. Curcumin was associated with improved collagen organization, increased collagen content, and enhanced biomechanical indices in rat Achilles tendon injury models across multiple studies [8,16,17]. Curcumin/Mg^2+^ hydrogel was linked to changes in fibrocartilage markers, collagen organization, and interface biomechanics in a tendon–bone model [5]. Procyanidin/Mg^2+^ hydrogel similarly showed matrix- and interface-associated effects in a tendon–bone context [27].

Quercetin-related tendinopathy evidence reported MMP and ICAM-1 expression changes alongside inflammatory and apoptotic markers in a collagenase-induced Achilles model [7]. Caffeic acid was associated with histologic and biomechanical improvements in a single Achilles tendon study [25]. Controlled-release curcumin influenced calcification-associated markers and tendon stem/progenitor cell differentiation in a tendon ectopic calcification model [12].

Across these studies, matrix endpoints are heterogeneous—spanning collagen isoform ratios, MMP expression, histologic scoring, and tensile strength—which limits direct cross-study comparison. However, the recurrence of matrix-associated signals across multiple compounds and models supports extracellular matrix regulation as a candidate pathway warranting standardized endpoint development.

### 4.4. Senescence-Associated Pathways

Quercetin was associated with reduced senescence/SASP markers in aged rat tendon stem/progenitor cells via AKT/NF-κB/NLRP3-mediated mitophagy [20], and a quercetin-containing aligned hydrogel was associated with improved tendon–bone interface indices in an osteoporotic rat model [18]. Quercetin also attenuated tendon degeneration indices in an Achilles tendon injury model [19].

In a separate line of evidence, D + Q reduced senescence markers and improved structural and biomechanical indices in aged rat rotator cuff models [21]. The most directly human-facing observation comes from a study demonstrating age-dependent increases in senescence markers in human rotator cuff tissue, with ex vivo D + Q exposure increasing COL1A1 expression [4]. This ex vivo finding provides human-tissue translational context for senescence biology but was not a patient intervention study.

Senescence-associated pathways are particularly relevant because they connect aging biology—a major clinical context for tendon and rotator cuff pathology—with measurable molecular targets. The availability of both animal and human tissue evidence for D + Q, alongside quercetin-only preclinical data, makes this one of the more developed mechanistic threads in the corpus.

### 4.5. Lineage Differentiation and Tissue-Interface Biology

Osteogenic, tenogenic, and angiogenic differentiation pathways appear prominently in tendon–bone interface studies. Icariin was associated with osteogenic and angiogenic markers in a rat rotator cuff reconstruction model [9]. Baicalein was linked to osteogenic differentiation and Wnt/β-catenin signaling in tendon–bone interface models [6]. TFRD promoted osteogenic differentiation and angiogenic support through MAPK/TGF-β-related signaling in ACL reconstruction contexts [23,24].

Curcumin-related evidence also intersects with differentiation biology. PI3K/Akt-associated tenogenic differentiation was reported in tendon injury models [17], while controlled-release curcumin attenuated aberrant osteogenic differentiation of tendon stem/progenitor cells in a calcification model [12]. These opposing effects—promoting tenogenic while suppressing inappropriate osteogenic differentiation—illustrate how compound effects may be context-dependent and pathway-specific.

### 4.6. Fatty Infiltration and Fibrosis in Rotator Cuff Models

Rotator cuff tear-associated fatty infiltration and fibrosis represent a distinct mechanistic domain. Tanshinone IIA suppressed fibro-adipogenic progenitor (FAP) differentiation and reduced fatty infiltration via Wnt/β-catenin signaling in a rat rotator cuff model [2]. Ginsenoside Rb1 modulated fibrosis, fat deposition, mitochondrial Ca^2+^-related markers, and SFRP1/Wnt signaling in rotator cuff tear models [3]. Tanshinone IIA was also studied in an Achilles tendon context using network pharmacology combined with in vivo validation [11].

These findings position fatty-infiltration biology as a potentially distinct translational pathway within tendon-adjacent tissue. However, both studies are single-compound, single-model investigations; replication and cross-compound comparison are needed before this pathway can be considered validated even at the preclinical level.

### 4.7. Mechanistic Integration

The current evidence base shows pathway convergence across inflammatory signaling, oxidative-stress response, extracellular matrix regulation, senescence-associated biology, lineage differentiation, and fatty-infiltration pathways. This convergence is notable because multiple compound classes show signals across overlapping pathways, suggesting that tendon and tendon–bone interface biology may be responsive to diverse bioactive inputs rather than requiring single-target specificity.

However, convergence at the pathway level does not imply therapeutic equivalence. Compounds, formulations, models, doses, and measured endpoints vary substantially, making it premature to rank compounds by efficacy or to identify a single optimal candidate. Figure 1 summarizes the conceptual pathway framework linking compounds to candidate mechanisms. The evidence-calibrated proximity assessment in Section 6 addresses which compound–pathway combinations have progressed furthest toward clinical relevance.

## 5. Domain-Specific Evidence: Tendon, Rotator Cuff, and Tendon-Bone Interface

### 5.1. Achilles Tendon and Tendon Remodeling

Achilles tendon injury and remodeling models constitute the largest subgroup. Curcumin improved histologic, biomechanical, and biochemical tendon healing indices across three independent rat studies [8,16,17]. Quercetin attenuated tendinopathy-associated pathology in a collagenase-induced model [7] and promoted healing in an Achilles tendon injury model [19]. Caffeic acid [25], ginsenoside Rg1 [10], Momordica charantia extract [30], and Hypericum/Tendoflex [29] were each evaluated in single rat Achilles tendon studies with varying endpoints. Tanshinone IIA appeared in both Achilles tendon [11] and adhesion-related models, with one study combining it with miR-29b inhibition [22]. Curcumin was also tested in formulation-specific variants, including PCL scaffolds [15] and nanomicelle/laser-assisted delivery [14].

The convergence of favorable structural and biomechanical indices across multiple compounds is notable, though endpoint, dose, formulation, and time-point heterogeneity limit direct comparison.

### 5.2. Rotator Cuff and Tendon–Bone Interface

Rotator cuff and tendon–bone interface models provide the second major evidence cluster. Icariin [9] and baicalein [6] were associated with osteogenic and angiogenic markers in tendon–bone interface models. TFRD was studied in ACL reconstruction and tendon–bone injury contexts, including BMSC-associated combination evidence [23,24]. Several biomaterial platforms were investigated in rotator cuff or tendon–bone settings: curcumin/Mg^2+^ hydrogel [5], procyanidin/Mg^2+^ hydrogel [27], quercetin-containing aligned hydrogel [18], and farnesol hydrogel membrane [28].

D + Q was tested in aged rotator cuff animal models with senescence/SASP marker changes and structural indices [21]. Protocatechuic acid was evaluated in a chronic rotator cuff fatty-degeneration model, though several outcomes were nonsignificant [26]. Tanshinone IIA [2] and ginsenoside Rb1 [3] were investigated in rotator cuff muscle fatty-infiltration and fibrosis models.

### 5.3. Human-Facing Translational Evidence

Only two Tier 2 sources were identified. The human rotator cuff tissue/ex vivo D + Q study reported age-associated senescence markers and selected biomarker changes after D + Q exposure, providing the most directly human-facing evidence in this corpus [4]. However, it did not administer D + Q as a patient intervention and did not assess pain, function, or surgical outcomes. The curcumin scoping review offers clinically oriented context for tendinopathy biology but represents contextual synthesis rather than primary efficacy evidence [13].

These sources inform translational gap analysis but do not convert the preclinical evidence base into clinical validation. The distance between current evidence and clinical application is addressed in the following section.

## 6. Translational Gaps, Nutritional Boundaries, and Future Directions

### 6.1. Bioavailability, Dietary Achievability, and Formulation Boundaries

A central constraint on translating the reviewed evidence is that the systemic and tissue-level exposure achieved by a plant-derived compound cannot be inferred from its name alone. Several of the most frequently studied compounds in this corpus are characterized by limited and variable oral bioavailability. Curcumin is well recognized to undergo poor intestinal absorption, rapid metabolism, and rapid systemic elimination, resulting in low circulating concentrations after oral administration [32]. Quercetin is extensively absorbed and metabolized through glucuronidation, sulfation, and methylation so that the predominant circulating species are conjugated metabolites rather than the aglycone typically applied in in vitro systems [33]. Because many studies in this corpus exposed cells or tissues directly or delivered compounds through local biomaterial systems, the parent-compound concentrations used experimentally may not correspond to the metabolite profiles or tissue concentrations achievable through oral intake. Tendon and tendon–bone interface exposure, therefore, cannot be assumed from systemic administration or from in vitro potency.

A related question is whether the exposures associated with favorable preclinical findings are achievable through habitual dietary intake. Effective concentrations in animal, in vitro, ex vivo, or local-delivery studies were frequently attained using purified compounds, highly administered doses, or sustained local release, conditions that differ substantially from the amounts and chemical forms obtained by consuming plant foods [33,34]. Habitual dietary intake, oral nutraceutical supplementation, purified pharmacological dosing, and local biomaterial delivery represent distinct exposure conditions, and dose equivalence across these conditions should not be assumed. Without comparable pharmacokinetic and tissue-distribution data, a mechanistic signal observed under one exposure condition cannot be transferred to another and, in particular, cannot be assumed to be reproducible through diet.

The corpus spans a wide range of exposure formats, including habitual dietary consumption, oral nutraceutical supplementation, purified compound administration, hydrogel, scaffold, nanomicelle, and membrane delivery, controlled-release local systems, ex vivo tissue exposure, and the dasatinib plus quercetin combination intervention. Each of these should be treated as a distinct translational entity rather than as interchangeable evidence for the underlying compound. A finding obtained with a curcumin-loaded hydrogel, for instance, characterizes that delivery platform and its local release behavior and does not establish that orally ingested curcumin would reach comparable tissue concentrations or produce comparable effects. Combination interventions such as D + Q likewise reflect the combined, and partly dasatinib-dependent, biology of the intervention and cannot be attributed to the plant-derived component alone.

These considerations define the nutritional relevance of the present review. That relevance lies not in supporting supplementation advice but in clarifying when extrapolation from preclinical plant-derived compound studies to dietary, functional-food, or nutraceutical recommendations is not justified. Because plant-derived compounds are frequently discussed in dietary and supplement contexts, whereas much of the tendon and tendon–bone evidence derives from preclinical, formulation-specific, or local-delivery systems, an explicit account of bioavailability, dietary achievability, and formulation boundaries is necessary to prevent premature nutraceutical positioning. In this sense, the review functions as a translational filter for the nutrition and nutraceutical community rather than as an endorsement of any specific compound or preparation.

### 6.2. Dose, Duration, and Model Heterogeneity

Dose, exposure duration, species, and outcome definitions vary substantially across the corpus. Several studies have dose or duration details requiring source-level verification [2,11,19,20]. Outcomes range from molecular markers to histologic scoring to biomechanical indices, without a shared endpoint framework. Future work should pair dose–response testing with pharmacokinetic and tissue-distribution measurements and adopt core outcome sets to enable cross-study comparison.

Network pharmacology studies can help prioritize candidate pathways, as demonstrated for tanshinone IIA [11], but predicted pathways require experimental validation before translational interpretation.

### 6.3. Safety, Interactions, and Natural-Product Misperception

Plant-derived origin should not be equated with clinical safety; the current corpus does not establish clinical, dose, interaction, or long-term safety in patients with tendon, rotator cuff, or tendon–bone interface disorders. The perception that botanical compounds are inherently safe is not supported by the reviewed evidence, which is predominantly preclinical and rarely characterizes adverse effects, dose thresholds, or drug interactions. These considerations are particularly relevant for older adults and patients receiving polypharmacy, in whom altered pharmacokinetics may modify both efficacy and risk.

Specific safety signals further illustrate this point. The curcumin–PCL scaffold study reported local aseptic inflammation [15], and dasatinib plus quercetin carries dasatinib-related safety and regulatory implications distinct from quercetin alone [4,21]. Mixed products such as Hypericum/Tendoflex complicate both attribution and safety interpretation [29]. Local biomaterial delivery systems additionally require evaluation of local tissue response, biocompatibility, and release-profile safety that is separate from the safety of the parent compound. Future translational designs should therefore incorporate prospective adverse-event reporting, interaction screening, and explicit attention to vulnerable populations.

### 6.4. Evidence-Calibrated Translational Proximity Map

Table 4 presents an evidence-calibrated map of translational proximity for compound–pathway–tissue combinations within the focused corpus. The map ranks relative proximity to human-facing validation within the reviewed evidence; it does not represent clinical readiness, therapeutic efficacy, or recommendation strength. Each combination was positioned according to explicit, evidence-type criteria rather than subjective judgment: (i) human-facing evidence—presence of human tissue, ex vivo, or clinically oriented evidence versus animal or in vitro evidence only; (ii) replication—whether findings recur across comparable models rather than only across heterogeneous formulations, routes, or endpoints; (iii) formulation clarity—whether the tested exposure is oral, local/biomaterial, ex vivo, or combination-specific; (iv) mechanism-endpoint linkage—whether the proposed pathway is connected to tissue- or interface-level outcomes; (v) safety and pharmacokinetic visibility—whether bioavailability, exposure, dose, and safety can be meaningfully interpreted; and (vi) claim boundary—the level of claim permitted by the assigned evidence tier. A higher position indicates greater proximity to human-facing validation within this corpus, not established patient benefit.

Most human-facing translational signals within the corpus. Dasatinib plus quercetin in rotator cuff senescence biology has human tissue/ex vivo evidence (Tier 2) demonstrating senescence-marker modulation in human rotator cuff tissue [4], supported by preclinical animal evidence of structural and biomechanical changes in aged models [21]. The next step is further human tissue validation, exposure–response assessment, and safety-focused early-phase translational study design before patient-intervention claims can be justified. This signal applies to D + Q as a combination and does not support quercetin-only clinical claims.

Recurrent preclinical signal across heterogeneous formulations. Curcumin in tendon remodeling and adhesion biology spans multiple independent animal studies with converging signals across inflammatory, matrix, and biomechanical endpoints [8,16,17]. However, the diversity of tested formulations—oral, hydrogel, nanomicelle, scaffold, and controlled-release—makes it unclear which preparation is the translational candidate. Formulation standardization and reproducibility testing are prerequisites to human-facing studies.

Emerging single-study signals. Icariin [9] and baicalein [6] at the tendon–bone interface, ginsenosides Rg1 [10] and Rb1 [3] in tendon and rotator cuff models, and tanshinone IIA in fatty-infiltration biology [2] are each supported by one or two preclinical studies. Independent replication in comparable models is needed before these compounds can be prioritized for translational development.

Exploratory or formulation dependent. Protocatechuic acid (nonsignificant outcomes noted) [26], farnesol hydrogel membrane [28], procyanidin/Mg^2+^ hydrogel [27], mixed products such as Hypericum/Tendoflex [29], and TFRD + BMSC combinations [24] remain preliminary, formulation-bound, or combination-specific. They identify candidate hypotheses but are not yet positioned for translational prioritization within the corpus.

### 6.5. Publication Bias and Selective Preclinical Positivity

Because the corpus is dominated by animal, in vitro, and mechanistic studies, the apparent convergence of favorable pathway-level findings may be influenced by publication bias and the selective availability of positive preclinical results. Negative or neutral studies are less likely to be published or indexed, and this underrepresentation can make convergent mechanistic signals appear stronger or more consistent than the underlying evidence warrants. This consideration reinforces that the proximity map reflects the distribution of available positive findings rather than a validated estimate of effect size or patient-facing translational value, and it underscores the need for preregistered, adequately powered preclinical and human-facing studies.

### 6.6. Toward Staged Validation

An evidence-calibrated translational pathway would proceed from reproducible formulations and validated exposure metrics through mechanism-linked preclinical endpoints to human tissue validation where appropriate and finally to controlled human studies with patient-centered outcomes and safety monitoring. The current corpus supports the first two stages; the third remains limited to a single ex vivo D + Q observation. Advancing beyond this point requires investment in standardized preparations, core outcome sets, phenotype-aware study designs, and prospective safety assessment.

Phenotype-specific study designs may also improve translational efficiency. Age, metabolic status, inflammatory burden, and tissue degeneration stage may influence biological response to candidate compounds. The prominence of senescence-related themes in quercetin and D + Q studies [4,20,21] and fatty-infiltration themes in rotator cuff models [2,3] suggests that matching compound mechanism to patient phenotype could be a productive design strategy for future studies.

### 6.7. Limitations of This Review

This review has several limitations. It was designed as a focused narrative synthesis rather than a systematic review; it is not PRISMA-compliant, did not apply formal risk-of-bias assessment, certainty grading, or meta-analysis, and does not claim exhaustive capture of the literature. Accordingly, the 31-article corpus should not be interpreted as an exhaustive dataset. The evidence base is predominantly preclinical and heterogeneous across species, tissue models, formulations, delivery routes, doses, exposure durations, and endpoints and may be affected by publication bias favoring positive preclinical findings. The evidence-tiering framework and the translational proximity map involve interpretive judgment; they were designed to make these judgments explicit, conservative, and reproducible at the level of evidence type and claim boundary, and they classify distance from patient-facing validation rather than internal validity or study quality. Several interventions involved local biomaterial delivery systems or combination approaches and therefore cannot be generalized to oral supplementation, dietary exposure, or single-compound effects, and safety data across the corpus are limited. The inclusion of dasatinib plus quercetin reflects the presence of a plant-derived quercetin component and human tissue-level evidence, but the findings remain combination-specific and cannot be attributed to quercetin alone. The pharmacokinetic references cited to support the dietary-translation discussion were used as contextual background and were not part of the 31-article core corpus or its tier distribution. Finally, the absence of robust clinical efficacy evidence precludes patient-facing treatment recommendations. These limitations are acknowledged not to diminish the synthesis but to delimit the claim boundaries that this review is intended to define.

## 7. Conclusions

Plant-derived bioactive compounds have been investigated across inflammatory, oxidative-stress, senescence-associated, matrix-regulatory, differentiation, and fatty-infiltration pathways in tendon and tendon–bone interface models. The current evidence base is predominantly preclinical, with no robust clinical efficacy evidence identified. Within this landscape, dasatinib plus quercetin in rotator cuff senescence biology represents the most human-facing translational signal within the current corpus, curcumin in tendon remodeling shows recurrent but formulation-fragmented preclinical support, and several other compounds remain at single-study or exploratory levels.

These compounds are best positioned as candidates for staged translational investigation rather than as established clinical interventions. By clarifying how far the current evidence has progressed along the translational pathway, this review defines where the boundary between biological plausibility and patient-facing validation should be drawn. The evidence-tiering framework, the evidence-calibrated translational proximity map, and the overclaim prevention checklist presented here may serve as practical tools for researchers navigating this boundary in tendon and tendon–bone interface biology. Accordingly, the current evidence should be used to guide hypothesis generation, formulation standardization, and staged validation, not to support clinical recommendations, supplementation advice, or patient-facing treatment claims.

## Figures and Tables

**Table 1:** Compound classes, evidence characteristics, and translational notes.

Compound/Class	Plant Source or Category	Key Pathways Investigated	Main Tissue/Model Domain	Evidence Tier	Translational Note	Supporting References
Curcumin	*Curcuma longa*	Inflammation, oxidative stress, tenogenic differentiation, matrix organization, adhesion biology, aberrant osteogenic differentiation	Achilles tendon injury/remodeling, tendon adhesion, tendon ectopic calcification, tendon–bone interface, formulation-specific delivery models	Tier 3; Tier 2 contextual review evidence	Recurrent across several tendon-related models and formulation contexts, but highly formulation-fragmented; standardization of preparation, route, and exposure is required before human-facing validation	[5,8,12,13,14,15,16,17]
Quercetin-only evidence	Dietary plants; source often not specified in experimental studies	Senescence/SASP, oxidative stress, inflammation, TSPC biology, matrix-associated responses	Achilles tendon injury/tendinopathy, aged tendon models, osteoporotic tendon–bone interface local-delivery models	Tier 3	Distinct from D + Q; quercetin-only and quercetin-formulation evidence should not be generalized to oral supplementation or D + Q biology	[7,18,19,20]
Dasatinib plus quercetin (D + Q)	Combination intervention; quercetin component is plant-derived, dasatinib is not	Senescence/SASP markers, COL1A1 expression, age-related rotator cuff tissue biology	Human rotator cuff tissue/ex vivo model; aged rotator cuff tendon–bone animal models	Tier 2 human tissue/ex vivo; Tier 3 animal	Most advanced translational signal in the corpus, but combination-specific; not attributable to quercetin alone and not yet patient-intervention evidence	[4,21]
Tanshinone IIA	*Salvia miltiorrhiza*	FAP differentiation, fatty infiltration, matrix remodeling, inflammation, adhesion-related biology	Rotator cuff fatty-infiltration models, Achilles tendon repair models, tendon adhesion models	Tier 3	Preclinical signal across tendon and rotator cuff-adjacent biology; combination evidence with miR-29b inhibition should not be attributed to Tanshinone IIA alone	[2,11,22]
Ginsenosides Rg1 and Rb1	*Panax ginseng*	Inflammatory signaling, tenocyte activity, apoptosis, fibrosis, fat deposition, mitochondrial Ca^2+^ signaling, Wnt/SFRP1-related pathways	Achilles tendinitis/tendon models; rotator cuff fibrosis and fatty-infiltration models	Tier 3	Single-study evidence per ginsenoside; biologically plausible but requires independent replication and standardized endpoints	[3,10]
Icariin	*Epimedium* species	Osteogenic and angiogenic markers, tendon–bone integration-associated biology	Rotator cuff tendon–bone interface animal model	Tier 3	Single animal study suggesting tendon–bone interface relevance; not sufficient for clinical or surgical outcome claims	[9]
Baicalein	*Scutellaria baicalensis*	Osteogenic differentiation, Wnt/β-catenin signaling	Tendon–bone interface animal model	Tier 3	Single preclinical tendon–bone interface signal; requires replication in comparable models	[6]
Total flavonoids of Rhizoma drynariae (TFRD)	*Drynaria fortunei*/Rhizoma drynariae	Osteogenic differentiation, MAPK/TGF-β signaling, BMSC-associated tendon–bone interface biology	ACL reconstruction tendon–bone interface models; BMSC-combination tendon–bone injury models	Tier 3	Mixed-extract evidence, including BMSC-combination context; findings should not be attributed to a single compound or to TFRD alone when combined with BMSCs	[23,24]
Phenolic acids: protocatechuic acid and caffeic acid	Various plant sources; caffeic acid from coffee and other plant foods	Antioxidant activity, inflammatory modulation, structural and biomechanical indices	Chronic rotator cuff fatty-degeneration model; Achilles tendon injury model	Tier 3	Limited single-study evidence; protocatechuic acid remains exploratory because several outcomes were nonsignificant	[25,26]
Procyanidins with magnesium ions	Plant procyanidins; specific plant source not consistently specified	Macrophage polarization, local release behavior, fibrocartilage/interface markers	Tendon–bone interface hydrogel model	Tier 3 biomaterial/local-delivery evidence	Formulation-specific hydrogel signal; not generalizable to oral procyanidins or procyanidin-only exposure	[27]
Farnesol	Plant essential oils and other natural sources	Cytokine modulation, collagen production, early structural tissue-response signals	Rotator cuff tear model using hydrogel membrane delivery	Tier 3 biomaterial/local-delivery evidence	Local hydrogel membrane evidence only; does not support farnesol supplementation or broad terpenoid-class claims	[28]
Botanical extracts and mixed products	*Hypericum perforatum*, Tendoflex^®^ components, *Momordica charantia*	Structural indices, collagen production, neovascularization, tenoblastic activity	Achilles tendon injury/remodeling models	Tier 3	Product- or extract-specific preclinical evidence; not convertible to single-ingredient claims	[29,30]

Note: Evidence tiers reflect the current manuscript corpus. Tier 1 indicates robust clinical efficacy evidence; Tier 2 indicates limited human-facing translational or clinically oriented evidence; Tier 3 indicates preclinical, mechanistic, animal, in vitro, biomaterial, or local-delivery evidence; and Tier 4 indicates indirect or limited-use evidence. References are provided at the row level to allow direct traceability of compound-specific evidence. D + Q was treated as a combination intervention and was not attributed to quercetin alone. Formulation-specific evidence involving hydrogels, scaffolds, nanomicelles, membranes, or controlled-release systems was interpreted according to the tested delivery platform.

**Table 2:** Evidence map by tissue/model domain.

Tissue/Model Domain	Representative Compounds/Formulations	Evidence Type	Evidence Tier	Key Findings	Translational Status	Supporting References
Achilles tendon injury and remodeling	Curcumin, quercetin, caffeic acid, ginsenoside Rg1, tanshinone IIA, *Momordica charantia*, Hypericum/Tendoflex, curcumin–PCL scaffold	Animal studies; animal + in vitro studies; biomaterial/local-delivery studies	Mainly Tier 3	Histologic, biochemical, biomechanical, inflammatory, oxidative-stress, tenogenic, and structural indices were reported across several rat Achilles tendon models	Largest preclinical evidence cluster; signals recur across compounds and models, but endpoint, dose, timing, and formulation heterogeneity limit direct comparison	[7,8,10,11,14,15,16,17,19,25,29,30]
Tendon adhesion and peritendinous fibrosis	Curcumin nanomicelles, curcumin-related local delivery, tanshinone IIA + miR-29b inhibition	Animal studies; animal + in vitro studies; local-delivery or combination-intervention studies	Tier 3	Adhesion-related indices, inflammatory markers, matrix remodeling, and tendon strength parameters were evaluated in rat tendon adhesion models	Mechanistically relevant to postoperative adhesion biology, but evidence remains formulation- or combination-specific	[14,17,22]
Tendon ectopic calcification and TSPC differentiation	Controlled-release curcumin	Animal + in vitro mechanistic evidence	Tier 3	Calcification-associated markers and inflammatory osteogenic differentiation of tendon stem/progenitor cells were investigated	Focused mechanistic signal; useful for aberrant differentiation biology, but not sufficient for calcific tendinopathy or clinical disease-modification claims	[12]
Rotator cuff tendon–bone interface and enthesis-related models	Curcumin/Mg^2+^ hydrogel, procyanidin/Mg^2+^ hydrogel, icariin, baicalein, TFRD, TFRD + BMSCs, quercetin-containing aligned hydrogel, farnesol hydrogel membrane	Animal studies; biomaterial/local-delivery studies; combination-intervention studies	Mainly Tier 3	Osteogenic differentiation, angiogenic markers, fibrocartilage/interface markers, macrophage polarization, collagen organization, mineralization, and biomechanical indices were reported	Central interface biology cluster; findings are promising for hypothesis generation but remain highly formulation-, model-, and endpoint-dependent	[5,6,9,18,23,24,27,28]
Rotator cuff senescence biology	D + Q; quercetin-containing local-delivery formulation	Human tissue/ex vivo evidence; aged animal models; biomaterial/local-delivery evidence	Tier 2 for human tissue/ex vivo D + Q; Tier 3 for animal/formulation studies	Age-associated senescence markers, SASP-related signals, COL1A1 expression, and tendon–bone structural indices were reported	Most human-facing translational signal within the corpus; D + Q should be interpreted as combination-specific and not as quercetin-only evidence	[4,18,20,21]
Rotator cuff muscle fatty infiltration and fibrosis	Tanshinone IIA, ginsenoside Rb1, protocatechuic acid	Animal studies; network pharmacology + in vivo validation; mechanistic rotator cuff models	Tier 3	FAP differentiation, Wnt/β-catenin signaling, fibrosis, fat deposition, mitochondrial Ca^2+^-related markers, and antioxidant endpoints were reported	Distinct tendon-adjacent muscle-remodeling cluster; biologically relevant but supported by limited single-compound or single-model evidence	[2,3,11,26]
Human-facing translational evidence	D + Q; curcumin scoping review context	Human rotator cuff tissue/ex vivo study; clinically oriented scoping review	Tier 2	Human rotator cuff tissue showed age-associated senescence biology with ex vivo D + Q-related biomarker changes; curcumin review provided tendinopathy-oriented clinical context	Provides translational context, not patient-intervention evidence; does not establish clinical efficacy, dosing, or surgical outcome benefit	[4,13]
Indirect or limited-use pathway background	Quercetin macrophage/microglia pathway evidence; general ACL tendon–bone strategy review	Indirect in vitro evidence; secondary review context	Tier 4	General inflammatory, oxidative-stress, or tendon–bone strategy context without direct plant-derived compound evidence in the target tendon/interface model	Should be used only as limited background; not core support for tendon, enthesis, rotator cuff, or clinical efficacy claims	[1,31]

Note: Evidence tiers reflect the current manuscript corpus. Tier 1 indicates robust clinical efficacy evidence; Tier 2 indicates limited human-facing translational or clinically oriented evidence; Tier 3 indicates preclinical, mechanistic, animal, in vitro, biomaterial, network pharmacology, or local-delivery evidence; and Tier 4 indicates indirect or limited-use evidence. D + Q, dasatinib plus quercetin; BMSC, bone marrow-derived mesenchymal stem cell; FAP, fibro-adipogenic progenitor; SASP, senescence-associated secretory phenotype; TFRD, total flavonoids of Rhizoma drynariae; TSPC, tendon stem/progenitor cell.

**Table 3:** Overclaim prevention checklist.

Scenario	Appropriate Claim Boundary	Supporting References
Animal model shows histologic, structural, or biomechanical improvement	Describe as “improved tissue-response indices in the tested animal model”; do not interpret as symptom relief, pain reduction, clinical functional recovery, reduced retear risk, or improved surgical outcome in patients.	[8,9,16,17,19,25]
Hydrogel, scaffold, nanomicelle, membrane, or controlled-release delivery is used	Interpret as a formulation-specific local-exposure finding; do not generalize to oral supplementation, dietary exposure, or compound-only effects.	[5,14,15,18,27,28]
Dasatinib plus quercetin (D + Q) is tested	Interpret as a combination-intervention signal; do not attribute findings to quercetin alone, and keep dasatinib-related safety and regulatory context visible.	[4,21]
Ex vivo human tissue biomarker change is observed	Interpret as translational biological relevance for the tested pathway; do not treat as patient treatment effect, dosing evidence, clinical efficacy, or surgical outcome evidence.	[4]
TFRD plus BMSC or other multi-component interventions are tested	Attribute findings to the full combination and model; do not assign the full effect to a single compound, extract, or plant-derived component alone.	[24]
Network pharmacology predicts candidate pathways	Treat as hypothesis-generating unless supported by experimental validation; do not describe predicted pathways as clinically validated mechanisms.	[11]
Mixed botanical or commercial product is evaluated	Interpret as product- or extract-specific evidence; do not convert the finding into a single-ingredient claim.	[29,30]
Some endpoints are nonsignificant or internally inconsistent	Treat the finding as exploratory and endpoint-dependent; avoid broad positive summary statements that ignore nonsignificant outcomes.	[26]
Animal behavioral or functional testing is reported	Describe as an animal behavioral or functional measure; do not equate it with patient-level physical performance, return to activity, or quality-of-life improvement.	[8,16,17,19]
Scoping review or narrative review is used as a source	Use as contextual synthesis; do not treat as primary efficacy evidence or as equivalent to randomized clinical evidence.	[1,13]
Local inflammation, biocompatibility concern, or safety signal is noted	Present as a safety issue requiring prospective evaluation; do not describe the intervention as clinically safe without appropriate safety data.	[15]
Multiple compounds show signals in the same pathway	Interpret as pathway convergence supporting biological plausibility; do not imply therapeutic equivalence, class effect, or clinical readiness across compounds.	[2,3,4,5,7,8,16,17,20,21]
A compound appears in several formulations or routes	Treat each formulation as a distinct translational entity; do not combine oral, hydrogel, scaffold, nanomicelle, ex vivo, and controlled-release evidence into a single compound-level claim.	[5,8,12,14,15,16,17]
Evidence is limited to a single animal or in vitro study	Classify as an emerging or exploratory signal; require independent replication before translational prioritization.	[6,9,10,25,26,28,30]
Human-facing evidence is absent	Limit interpretation to mechanistic plausibility or translational hypothesis; do not make patient-facing recommendations.	General principle based on tier framework

Note: This checklist summarizes interpretive boundaries applied throughout the review. Supporting references are representative rather than exhaustive for each scenario. D + Q, dasatinib plus quercetin; BMSC, bone marrow-derived mesenchymal cell; TFRD, total flavonoids of Rhizoma drynariae. Findings from animal, in vitro, biomaterial, local-delivery, or ex vivo studies were interpreted according to evidence tier, model, formulation, route, and endpoint type.

**Table 4:** Evidence-calibrated translational proximity map within the focused corpus.

Relative Translational Proximity Level	Compound–Pathway–Tissue Combination	Current Evidence Position	Interpretation	Priority Next Step	Supporting References
Most human-facing translational signal within the corpus	D + Q → rotator cuff senescence biology	Tier 2 human rotator cuff tissue/ex vivo evidence; Tier 3 aged rotator cuff animal evidence	Most human-facing signal in the corpus. Supports senescence biology as a translationally relevant pathway in rotator cuff tissue but remains combination-specific and not attributable to quercetin alone.	Further human tissue validation, exposure–response assessment, and safety-focused early-phase translational study design	[4,21]
Recurrent preclinical signal across heterogeneous formulations	Curcumin → tendon remodeling, adhesion biology, and tendon matrix regulation	Multiple Tier 3 animal studies; several formulation variants, including oral exposure, hydrogel, nanomicelle, scaffold, and controlled-release systems	Recurrent tendon-related signal across inflammatory, oxidative-stress, matrix, biomechanical, adhesion, and differentiation endpoints; the translational candidate remains unclear because the evidence is formulation-fragmented.	Formulation standardization, reproducibility testing, exposure validation, and dose–response assessment before human-facing studies	[5,8,12,14,15,16,17]
Developed preclinical senescence signal	Quercetin-only evidence → aged tendon/tendon stem-progenitor cell senescence	Tier 3 aged tendon, tendinopathy, and tendon stem/progenitor-cell evidence	Supports quercetin-related senescence, oxidative-stress, and mitophagy-associated hypotheses in preclinical tendon models; should remain separate from D + Q evidence.	Independent replication, route-specific exposure testing, and separation of quercetin-only versus combination-intervention designs	[7,18,19,20]
Interface-focused emerging signal	Icariin/baicalein → tendon–bone interface osteogenic and angiogenic signaling	Tier 3 animal tendon–bone interface studies	Suggests osteogenic, angiogenic, and Wnt/β-catenin-associated interface biology, but currently rests on limited single-study-level evidence for each compound.	Independent replication in comparable tendon–bone interface models using standardized histologic, biomechanical, and molecular endpoints	[6,9]
ACL/tendon–bone interface emerging signal	TFRD ± BMSC → tendon–bone interface differentiation and remodeling	Tier 3 ACL reconstruction and tendon–bone injury/interface models; includes combination evidence	Supports interest in osteogenic differentiation and MAPK/TGF-β-related interface biology, but mixed-extract and BMSC-combination designs limit attribution.	Replication of TFRD-only and TFRD + BMSC designs separately; clearer attribution of compound, extract, and cellular co-intervention effects	[23,24]
Muscle-remodeling emerging signal	Tanshinone IIA → rotator cuff fatty infiltration/FAP differentiation	Tier 3 rotator cuff fatty-infiltration model; network pharmacology plus in vivo Achilles tendon validation	Suggests relevance to tendon-adjacent muscle degeneration, FAP biology, inflammation, and Wnt/β-catenin signaling; evidence remains single-model or pathway-prediction dependent.	Replication in rotator cuff fatty-infiltration models, dose/duration clarification, and validation of tissue-specific functional endpoints	[2,11,22]
Tendon/rotator cuff remodeling emerging signal	Ginsenosides Rg1 and Rb1 → tendon inflammation, tenocyte activity, fibrosis, and fatty infiltration	Tier 3 single-study evidence per ginsenoside	Biologically plausible preclinical signals in Achilles tendinitis and rotator cuff fibrosis/fatty-infiltration models; no replicated compound-specific evidence yet.	Independent replication with standardized inflammatory, matrix, mitochondrial, fibrosis, and fatty-infiltration endpoints	[3,10]
Exploratory antioxidant/structural signal	Protocatechuic acid and caffeic acid → rotator cuff fatty degeneration or Achilles tendon structural indices	Tier 3 single-study evidence; protocatechuic acid includes nonsignificant endpoints	Exploratory signal involving antioxidant and structural endpoints; interpretation should remain endpoint-dependent.	Pilot-level replication and clearer endpoint hierarchy before translational prioritization	[25,26]
Formulation-bound exploratory signal	Farnesol membrane and procyanidin/Mg^2+^ hydrogel → local delivery in rotator cuff or tendon–bone interface models	Tier 3 biomaterial/local-delivery studies	Local-exposure signal tied to the tested biomaterial platform; not generalizable to supplementation or compound-only effects.	Formulation reproducibility, release-profile validation, local safety assessment, and comparison against platform controls	[27,28]
Product- or combination-specific exploratory signal	Hypericum/Tendoflex, Momordica charantia, and other mixed botanical products → Achilles tendon remodeling	Tier 3 single-product or extract-specific animal studies	May support product-specific hypotheses but cannot be converted into single-compound claims.	Product standardization, ingredient attribution, independent replication, and safety profiling	[29,30]

Note: This table ranks compound–pathway–tissue combinations by translational proximity within the current manuscript corpus, not by clinical efficacy. “Most human-facing translational signal within the corpus” indicates the presence of human tissue or ex vivo evidence, not established patient benefit. “Recurrent preclinical signal across heterogeneous formulations” indicates convergence across more than one preclinical study or formulation context and should be interpreted as recurrence across heterogeneous formulations and tendon-related models rather than replication in directly comparable models. “Emerging signal” indicates limited preclinical evidence requiring independent replication. “Exploratory” indicates single-study, formulation-bound, nonsignificant, product-specific, or attribution-limited evidence. D + Q, dasatinib plus quercetin; BMSC, bone marrow-derived mesenchymal stem cell; FAP, fibro-adipogenic progenitor; TFRD, total flavonoids of Rhizoma drynariae.

## Data Availability

No new data were generated or analyzed in this study. Data sharing is not applicable to this article.

## Supplementary Materials

The following supporting information can be downloaded at: https://www.mdpi.com/article/10.3390/nu18132120/s1, Figure S1: Corpus-prioritization diagram; Table S1: Structured extraction table for the 31-article corpus.

## Abbreviations

Abbreviation	Meaning
ACL	Anterior cruciate ligament
BMSC	Bone marrow-derived mesenchymal stem cell
COL1A1	Collagen type I alpha 1 chain
D + Q	Dasatinib plus quercetin
ECM	Extracellular matrix
FAP	Fibro-adipogenic progenitor
ICAM-1	Intercellular adhesion molecule 1
MDA	Malondialdehyde
MMP	Matrix metalloproteinase
NF-κB	Nuclear factor kappa B
NLRP3	NLR family pyrin domain containing 3
PCL	Polycaprolactone
PCA	Protocatechuic acid
PI3K/Akt	Phosphoinositide 3-kinase/protein kinase B
RC	Rotator cuff
RCT	Randomized controlled trial
ROS	Reactive oxygen species
SASP	Senescence-associated secretory phenotype
SFRP1	Secreted frizzled-related protein 1
SOD	Superoxide dismutase
TFRD	Total flavonoids of Rhizoma drynariae
TGF-β	Transforming growth factor beta
TSPC	Tendon stem/progenitor cell
