# Supplementary figures and images for: Plant-Derived Bioactives in Tendon and Enthesis Biology: An Evidence-Tiered Narrative Review
Source: Nutrients. 2026 Jun 30;18(13):2120. doi: 10.3390/nu18132120 (PMC13364093; doi:10.3390/nu18132120)

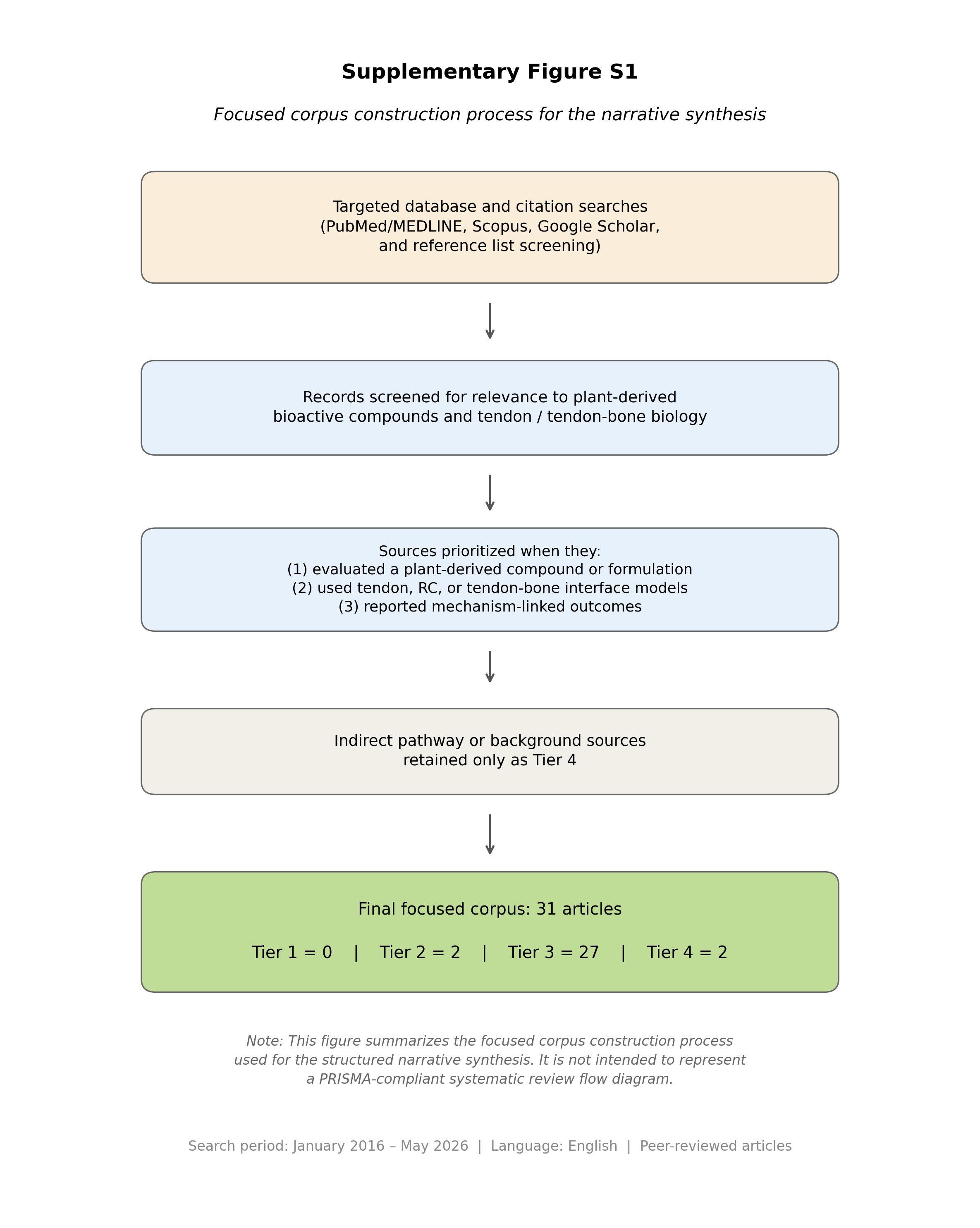

Supplement: Supplementary file 1 [file nutrients-18-02120-s001.zip › Supplementary_Figure_S1.png]
